# General population normative values for the EORTC QLQ-C30 by age, sex, and health condition for the French general population

**DOI:** 10.1186/s41687-024-00719-7

**Published:** 2024-05-02

**Authors:** Micha J. Pilz, Fanny L.C. Loth, Sandra Nolte, Anna M.M. Thurner, Eva-Maria Gamper, Amélie Anota, Gregor Liegl, Johannes M. Giesinger, on behalf of the EORTC Quality of Life Group

**Affiliations:** 1 Health Outcomes Research Unit, University Hospital of Psychiatry II, Medical University of Innsbruck, Innrain 43, Innsbruck, 6020 Austria; 2 Psychological Diagnostics and Intervention, Catholic University of Eichstätt-Ingolstadt, Eichstätt, Germany; 3 Department of Psychosomatic Medicine, Charité – Universitätsmedizin Berlin, Corporate Member of Freie Universität Berlin and Humboldt-Universität zu Berlin, Berlin, Germany; 4 Department of Nuclear Medicine, Medical University of Innsbruck, Innsbruck, Austria; 5 Department of Clinical Research and Innovation and Human and Social Sciences Department, Centre Léon Bérard, Lyon, France; 6 Melbourne Health Economics, Centre for Health Policy, Melbourne School of Population and Global Health, The University of Melbourne, Melbourne, Australia

**Keywords:** EORTC QLQ-C30, France, Normative values, General population, Health-related quality of life, Comorbidity

## Abstract

**Background:**

General population normative values for the widely used health-related quality of life (HRQoL) measure EORTC QLQ-C30 support the interpretation of trial results and HRQoL of patients in clinical practice. Here, we provide sex-, age- and health condition-specific normative values for the EORTC QLQ-C30 in the French general population.

**Methods:**

French general population data was collected in an international EORTC project. Online panels with quota samples were used to recruit sex and age groups. Number and type of comorbidities were assessed. Descriptive statistics were used to calculate general population values for each QLQ-C30 scale, separately for sex, age, and presence of one- and more chronic health conditions. A multivariate linear regression model has been developed to allow estimating the effect of sex, age, and the presence for one- and more chronic health conditions on EORTC QLQ-C30 scores. Data was weighted according to United Nation statistics adjusting for the proportion of sex and age groups.

**Results:**

In total, 1001 French respondents were included in our analyses. The weighted mean age was 47.9 years, 514 (51.3%) participants were women, and 497 (52.2%) participants reported at least one health condition. Men reported statistically significant better scores for Emotional Functioning (+9.6 points, *p* = 0.006) and Fatigue (−7.8 point; *p* = 0.04); women reported better profiles for Role Functioning (+8.7 points; *p* = 0.008) and Financial Difficulty (−7.8 points, *p* = 0.011). According to the regression model, the sex effect was statistically significant in eight scales; the effect of increasing age had a statistically significant effect on seven of the 15 EORTC QLQ-C30 scales. The sex- and age effect varied in its direction across the various scales. The presence of health conditions showed a strong negative effect on all scales.

**Conclusion:**

This is the first publication of detailed French normative values for the EORTC QLQ-C30. It aims to support the interpretation of HRQoL profiles in French cancer populations. The strong impact of health conditions on QLQ-C30 scores highlights the importance of considering the impact of comorbidities in cancer patients when interpreting HRQoL data.

**Supplementary Information:**

The online version contains supplementary material available at 10.1186/s41687-024-00719-7.

## Introduction

The patient’s perspective and its standardized assessment via patient-reported outcome (PRO) measures are key aspects in the evaluation of cancer treatments. This is evident not only in the large number of well-validated PRO measures available but it is also reflected in international guidelines on how to incorporate PRO measures in clinical trials [1, 2] and daily practice [3]. The increasing use of PROs in daily clinical practice [4–6] and their widespread implementation as study endpoints in cancer clinical trials [7, 8] support this statement.

The European Organization for Research and Treatment of Cancer Quality of Life Questionnaire Core-30 (EORTC QLQ-C30) [9] is the most widely used PRO measure in cancer clinical trials [10, 11] and clinical practice [6]. To date, the EORTC QLQ-C30 has been translated into over 100 languages and its use is reported in more than 5000 publications indexed on PubMed alone. Various efforts have been made to improve interpretability of its 15 scales for the multidimensional assessment of health-related quality of life (HRQoL). Minimal important differences have been published to guide interpretation of differences between time points or patient groups [12, 13], the development of thresholds for clinical importance supports interpretation of scores from individual patients at a single-time point [14, 15], and the publication of general population normative data provides important comparative information for interpretation of scores from cancer patients [16–20].

Such normative data, obtained from the general population, provide a reference score against which the scores of individual patients or patient groups can be compared. Normative data typically provide reference values for the whole target population (e.g., general population in a specific country) and commonly for groups defined by age and sex. For the EORTC QLQ-C30, country-, sex-, and age-specific values have been reported previously, whereby the impacts of economic factors [21, 22] or comorbidities on the EORTC QLQ-C30 reference values have been highlighted in many publications [18, 22–28].

A large number of comorbidities is not only associated with worse prognosis regarding overall survival [29–32] but has also a strong negative impact on HRQoL [33, 34]. Notably, the treatment experience of patients with cancer and their HRQoL are likely influenced by comorbidities and not just the main cancer diagnosis [34]. Due to higher complication rates, lower treatment tolerability, or presence of polypharmacy, comorbidities contribute to worse health outcomes in general, and higher healthcare costs in patients with cancer [34–36]. Furthermore, patients with cancer and multimorbidity are more likely to receive modified treatment [32] or treatment without curative intent [34]. Approximately 40% of patients with cancer have at least one additional (chronic) condition whereas 15% have two or more conditions. The most common comorbidities among these patients include cardiovascular diseases, obesity, metabolic illness, mental health problems, and musculoskeletal conditions [37]. Thus, general population normative data that display the influence of comorbidities on HRQoL can provide valuable complementary information for clinicians and researchers.

In this article, we present detailed normative data for the EORTC QLQ-C30 for the French general population relying on data from a previous project [16]. Adding to this previous publication, we present detailed results for the French population with normative data for groups defined by sex, age, and the presence of health conditions. To the best of our knowledge, this is the first manuscript reporting detailed EORTC QLQ-C30 normative data for the French general population by sex and age, thus enhancing the interpretability of EORTC QLQ-C30 data for French cancer patients. These normative data may be used in daily clinical practice [38]; in cancer clinical trials [39] assessing French patients; or for benchmarking in French hospitals.

## Methods

### EORTC QLQ-C30 questionnaire

The EORTC QLQ-C30 [9] is a standardized and well-validated [40] HRQoL questionnaire for patients with cancer. The EORTC QLQ-C30 consists of 30 items that assess five functioning dimensions (physical, social, role, emotional, and cognitive), nine symptoms (fatigue, pain, nausea/vomiting, dyspnoea, sleep disturbances, appetite loss, diarrhoea, constipation, and financial difficulties), and Global health status/Quality of life (QOL). The recall period for all but the physical functioning scale is one week (no recall period for physical functioning) [9]. Each item is scored on a 4-point ordinal scale except the two last items on a 1 to 7 scale and summarized into the 15 HRQoL dimensions according to the EORTC QLQ-C30 scoring manual [41]. High scores (range 0–100) on the symptom scales indicate a high symptom burden whereas high scores for the functioning and Global health status/QOL scales indicate high HRQoL. The EORTC QLQ-C30 Summary Score aggregates the information gathered from all individual scales (with the exception of Global health status/QOL and Financial Impact) into one singular overall result [42, 43]. Please note that for the Summary Score the scoring direction of the symptom scales was reversed, so that a high Summary Score corresponds to high overall HRQoL.

### Data collection

The data in this manuscript stem from a multinational EORTC project assessing the general population of 13 European countries, Canada, and the United States. Data collection was carried out by the panel research company GfK SE (https://www.gfk.com) that contacted their panel members for participation in this study. Participants completed a total of 86 items of the EORTC item banks [44], including the 30 items of the EORTC QLQ-C30, and answered questions assessing sociodemographic characteristics as well as presence of doctor-diagnosed chronic conditions [16, 45]. The selection of chronic conditions was based on their prevalence in the community (i.e., chronic pain, heart disease, cancer, depression, chronic obstructive pulmonary disease, arthritis, diabetes, asthma, anxiety disorder, obesity, drug/alcohol use disorder), including a free-text option for respondents to add any further chronic condition(s) not included in the list. This analysis relies on the responses from French participants, with quota sampling to obtain 100 patients for each of the groups defined by age (18–39, 40–49, 50–59, 60–69 and ≥70 years) and sex. Data was collected in March and April of 2017 and only complete data sets were eligible for the analysis. The panel research company GfK SE claims that the response rate of internet panels is between 75 and 90% [16]. No further information on response rates and drop-out rates was made available.

### Statistical analysis

We weighted the collected data to match the world population distribution statistics as published by the United Nations (UN) in 2017 [46], which were the most recent statistics available at the time of data collection. Weights were calculated to adjust for under-and overrepresentation of quotas in the sample and ranged from 0.647 (for men ≥ 70 years) to 3.576 (for men in the age group 18–39 years). Sample characteristics are provided for weighted and unweighted data. Relying on the weighted data, general population normative values are presented as mean and standard deviation (SD), for groups defined according to sex, age (18–39, 40–49, 50–59, 60–69 and ≥70 years), and existing health conditions (none vs. one and more). Ceiling and floor effects were calculated for the EORTC QLQ-C30 scales, displaying the percentage of participants obtaining the highest or the lowest possible score, respectively. Additionally, we established a multivariate linear regression model estimating the effect of sex, of age (continuous variable with linear and quadratic term), the age by sex- interaction, and the presence of comorbidities (none, one or more) for each EORTC QLQ-C30 scale. The multivariate regression model aims to allow a precise calculation of normative values for the French population and to supplement the descriptive normative data tables. The selection of covariates was consistent with previously applied methods [18–20], whereby the full model (block entry) retained all covariates in the model. To allow for a non-linear association between age and the QLQ-C30 scores, the regression model included a quadratic age term as well as a linear age term. IBM SPSS version 21 was used for the statistical analysis.

## Results

In the unweighted sample of 1001 French respondents, 499 participants (49.9%) were women, and the mean age was 53.6 (SD 14.7) years. Applying weights based on UN statistics [46] increased the proportion of women to 51.3% and decreased the mean age to 47.9 (SD 17.0) years. In the weighted sample, 42.7% reported having a postgraduate degree, 66.0% reported being married or in a steady relationship, and 44.0% were working full-time. Having one or more health conditions was reported by 52.2% of participants. The statistical weights applied to the data from individual participants ranged from 0.647 to 3.576. See Table 1 for unweighted and weighted sample characteristics.

**Table 1:** Sample characteristics (N = 1001)

		Unweighted data	Weighted data
Sex N (%)	Male	502 (50.1%)	487 (48.7%)
	Female	499 (49.9%)	514 (51.3%)
Age	M (SD)	53.6 (14.7)	47.9 (17.0)
	Median [IQR]	54 (25)	47 (30)
Education N (%)	Less than compulsory education	1 (0.1%)	1 (0.1%)
	Compulsory school	60 (6.1%)	51 (5.2%)
	Some post-compulsory school	164 (16.7%)	135 (13.7%)
	Post-compulsory below university	105 (10.7%)	112 (11.4%)
	University degree (Bachelor)	277 (28.2%)	263 (26.8%)
	Postgraduate degree	377 (38.3%)	419 (42.7%)
	Prefer not to answer	17	19
Marital status N (%)	Single/not in steady relationship	154 (15.5%)	208 (21.1%)
	Married or in a steady relationship	682 (68.8%)	651 (66.0%)
	Separated/divorced/widowed	155 (15.6%)	128 (13.0%)
	Prefer not to answer	10	14
Employment status N (%)	Full-time employed	388 (39.1%)	437 (44.0%)
	Part-time employed	74 (7.5%)	73 (7.3%)
	Self-employed	25 (2.5%)	25 (2.5%)
	Student	17 (1.7%)	50 (5.0%)
	Unemployed	67 (6.7%)	80 (8.0%)
	Retired	368 (37.1%)	283 (28.5%)
	Homemaker	39 (3.9%)	34 (3.4%)
	Other	15 (1.5%)	12 (1.2%)
	Prefer not to answer	8	7
Comorbidity N (%)	None	402 (42.3%)	456 (47.8%)
	One health condition	301 (31.7%)	267 (28.0%)
	Two-to-Three health conditions	216 (22.7%)	204 (21.4%)
	Four and more health conditions	31 (3.3%)	26 (2.8%)
	Chronic pain	275 (28.9%)	259 (27.1%)
	Heart disease	66 (6.9%)	54 (5.7%)
	Cancer	28 (2.9%)	24 (2.6%)
	Depression	57 (6.0%)	54 (5.6%)
	COPD	29 (3.1%)	28 (2.9%)
	Arthritis	39 (4.1%)	34 (3.6%)
	Diabetes	73 (7.7%)	58 (6.1%)
	Asthma	50 (5.3%)	59 (6.2%)
	Anxiety disorder	112 (11.8%)	108 (11.4%)
	Obesity	68 (7.2%)	61 (6.4%)
	Drug/alcohol disorder	10 (1.1%)	9 (0.9%)
	Other	145 (15.3%)	123 (12.9%)
	Prefer not to answer	40	39
	Missing	11	9

In Table 2, normative data are presented for the total sample and for specific age groups. For the overall sample, mean scores on the functioning scales were 89.1 (SD 15.9) for Physical Functioning, 90.5 (SD 20.8) for Social Functioning, 87.8 (SD 22.4) for Role Functioning, 76.7 (SD 24.3) for Emotional Functioning, and 86.7 (SD 19.5) for Cognitive Functioning. For the functioning scales, the highest mean difference between age groups was found for Emotional Functioning in the age group 18–39 years as compared with the group aged 70+ years (mean difference of +11.4 points, indicating better functioning status for the older group) and Social Functioning in the age group 60–69 years as compared with those aged 70+ years (mean difference of +11.0 points, indicating better functioning status for the older group). For the symptom scales, the highest mean difference between age groups was found for Fatigue in the age group 18–39 years compared with age 60–69 years (mean difference of −16.7 points, indicating lower symptom burden for the older group) and for Insomnia in the age group 50–59 years compared with age 70+ years (mean difference of −8.7 points, indicating lower symptom burden for the older group). The highest mean value for the QLQ-C30 Summary Score (88.2 points; SD 12.3) was found for the age group 60–69 years, a finding that was mirrored by the highest Global health status/QOL score (69.8 points; SD 17.7) in this age group. The results of sex- and age group-specific analysis is provided in Supplementary Table S1.

**Table 2:** EORTC QLQ-C30 reference values for the general population of France (weighted data)

	All		18–39 years		40–49 years		50–59 years		60–69 years		≥70 years	
	Mean	SD	Mean	SD	Mean	SD	Mean	SD	Mean	SD	Mean	SD
Physical functioning	89.1	15.9	90.4	16.8	89.5	16.1	88.7	16.0	88.9	14.8	86.2	14.2
Role functioning	87.8	22.4	89.5	22.2	87.8	22.8	85.4	23.8	88.7	21.7	85.6	21.7
Emotional functioning	76.7	24.3	72.0	27.4	74.3	25.0	78.0	22.9	82.4	20.2	83.4	17.1
Cognitive functioning	86.7	19.5	86.5	21.6	84.9	21.1	86.0	20.3	90.0	14.6	86.8	15.0
Social functioning	90.5	20.8	90.2	21.9	87.8	22.5	89.3	21.5	82.1	18.7	93.1	16.4
Global health status/QOL	68.2	20.1	69.7	19.7	65.3	20.9	66.5	22.5	69.8	17.7	67.8	19.6
Fatigue	27.7	26.2	34.7	27.7	28.2	26.1	27.5	26.1	18.0	22.5	20.2	21.0
Nausea/vomiting	4.1	13.5	6.2	17.1	5.1	15.4	3.2	10.4	2.1	9.1	1.2	4.7
Pain	19.6	24.7	18.2	24.3	17.7	24.6	23.6	28.2	18.4	23.3	21.8	23.0
Dyspnoea	14.4	23.8	13.2	22.5	13.0	23.8	13.8	23.9	15.2	24.7	17.3	25.5
Insomnia	25.9	30.6	25.9	31.7	29.3	30.9	30.1	32.0	22.7	29.0	21.4	26.6
Appetite loss	8.0	19.7	10.7	23.2	8.8	20.7	8.0	19.3	4.1	14.1	4.5	12.9
Constipation	11.1	21.2	10.9	22.2	11.2	21.5	10.8	20.6	10.4	19.1	12.4	21.3
Diarrhoea	7.3	18.8	8.8	20.2	8.8	21.3	7.0	18.5	5.0	14.9	4.7	15.6
Financial problems	6.7	19.3	7.1	19.2	10.5	25.6	7.0	20.1	3.2	11.4	4.5	15.9
QLQ-C30 Summary score	85.6	15.1	84.6	16.8	84.8	16.4	84.9	14.9	88.2	12.3	87.0	11.8

In the French general population, the EORTC QLQ-C30 Summary Score had a ceiling effect of 9.0%, indicating that nearly one in ten participants reported the highest possible functioning levels and no symptoms for the scales included in this score over the recall period of 1 week. On the functioning scales, ceiling effects were most pronounced for Social, Role, and Cognitive Functioning, with 75.4%, 68.6%, and 54.9% of participants respectively reporting no impairment. On the symptom scales, floor effects (i.e., a lack of symptoms) were most pronounced in the scales Nausea/Vomiting (87.2% of participants reporting no problem), Financial Difficulties (86.6%), Diarrhoea (83.5%), and Appetite Loss (82.5%). For further information on floor and ceiling effects see Table 3.

**Table 3:** Floor and ceiling effects in the EORTC QLQ-C30 scales (weighted data)

	Lowest possible score	Highest possible score
	(0 points) N (%)	(100 points) N (%)
Physical functioning	3 (0.3%)	428 (42.8%)
Role functioning	17 (1.7%)	687 (68.6%)
Emotional functioning	13 (1.3%)	289 (28.8%)
Cognitive functioning	10 (1.0%)	550 (54.9%)
Social functioning	13 (1.3%)	755 (75.4%)
Global health status/QOL	6 (0.6%)	77 (7.7%)
Fatigue	256 (25.6%)	31 (3.1%)
Nausea/vomiting	872 (87.2%)	6 (0.6%)
Pain	462 (46.1%)	32 (3.2%)
Dyspnoea	675 (67.5%)	26 (2.6%)
Insomnia	490 (48.9%)	67 (6.7%)
Appetite loss	826 (82.5%)	19 (1.9%)
Constipation	741 (74.0%)	19 (1.9%)
Diarrhoea	836 (83.5%)	19 (1.9%)
Financial problems	867 (86.6%)	21 (2.1%)
QLQ-C30 summary score	0 (0.0%)	90 (9.0%)

The multivariable linear regression model (Table 4) revealed the influence of sex, age, and self-reported health conditions on the EORTC QLQ-C30 scales. Adjusted R^2^ for the model ranged from 0.027 for Diarrhoea to 0.215 for Global health status/QOL. The influence of sex varied across scales. Whilst male sex was significantly associated with worse scores on the Role Functioning scale (−8.64 points, *p* = 0.001), the opposite was observed for the Emotional Functioning (+12.35 points, *p* ≤ 0.001) and Cognitive Functioning (+4.90 points, *p* = 0.042) scales. Additionally, men reported lower Insomnia (−13.38, *p* ≤ 0.001) and lower Fatigue (−7.79 points, *p* = 0.012) than women, but higher scores for Financial difficulties (+6.68 points, *p* = 0.006) and Diarrhoea (+5.43 points, *p* = 0.025). Increasing age was associated with better HRQoL in the French population. The single or quadratic age terms were significantly associated with lower scores for Fatigue, Dyspnoea, Insomnia or Financial Difficulties. Additionally, the single or quadratic age term was significantly associated with higher scores for Social Functioning and Global health status/QOL. Finally, we found a strong effect for the presence of self-reported comorbidities on the EORTC QLQ-C30 scales and Summary Score. The effect of reporting one or more health conditions was associated with an increase of up to +21.78 points (*p* ≥ 0.001) for Pain and +19.44 points (*p* ≤ 0.001) for Fatigue on the symptom scales. Furthermore, on the functioning scales, the presence of health conditions was associated with lower scores, e.g., −17.76 points (*p* < 0.001) for Role Functioning, and −15.06 points (*p* < 0.001) for Emotional Functioning. A similar pattern of lower scores was observed for the Global health status/QOL scale and the Summary Score. Supplementary Table S2 displays the EORTC QLQ-C30 mean values for sex and age groups, with or without a self-reported health condition. Participants with self-reported health conditions had worse HRQoL than participants without self-reported health conditions across all HRQoL domains. Table S3 displays the incremental impact of an increasing number of comorbidities on EORTC QLQ-C30 scale scores.

**Table 4:** Regression models for the EORTC QLQ-C30 values in general population of France

	Intercept	Sex^a^		Age^b^		Age squared^c^		Age-by-sex^d^		Health condition^e^		Adj. R^2^
		Coeff.	*p*-value	Coeff.	*p*-value	Coeff.	*p*-value	Coeff.	*p*-value	Coeff.	*p*-value	
Physical functioning	92.14	−0.84	0.658	0.27	0.028	0.00	0.022	0.07	0.244	−12.33	<0.001	0.146
Role functioning	100.69	−8.64	0.001	−0.13	0.439	0.00	0.616	0.22	0.004	−17.76	<0.001	0.153
Emotional functioning	68.23	12.35	<0.001	0.29	0.119	0.00	0.315	−0.15	0.079	−15.06	<0.001	0.151
Cognitive functioning	87.63	4.90	0.042	0.12	0.421	0.00	0.611	−0.11	0.110	−13.34	<0.001	0.110
Social functioning	98.35	−1.39	0.587	−0.34	0.037	0.01	0.003	0.10	0.160	−14.37	<0.001	0.118
Global health status/QOL	76.78	6.12	0.009	−0.32	0.032	0.01	0.001	−0.09	0.207	−18.41	<0.001	0.215
Fatigue	37.62	−7.79	0.012	−0.61	0.002	0.00	0.766	0.06	0.491	19.44	<0.001	0.186
Nausea/vomiting	3.35	2.95	0.082	−0.04	0.720	0.00	0.476	−0.09	0.079	5.81	<0.001	0.058
Pain	10.62	0.44	0.882	0.00	0.993	0.00	0.742	−0.08	0.306	21.78	<0.001	0.177
Dyspnoea	13.68	−4.91	0.097	−0.39	0.040	0.00	0.138	0.13	0.128	14.79	<0.001	0.091
Insomnia	18.68	−13.38	<0.001	0.74	0.002	−0.02	<0.001	0.15	0.179	18.89	<0.001	0.132
Appetite loss	8.64	−0.27	0.913	−0.09	0.589	0.00	0.458	−0.02	0.739	8.94	<0.001	0.057
Constipation	7.54	0.60	0.824	0.09	0.611	0.00	0.719	−0.14	0.077	8.49	<0.001	0.048
Diarrhoea	4.63	5.43	0.025	0.03	0.868	0.00	0.511	−0.14	0.054	5.76	<0.001	0.027
Financial difficulties	−2.06	6.68	0.006	0.39	0.012	−0.01	0.002	−0.16	0.024	9.13	<0.001	0.065
QLQ-C30 summary score	87.88	1.89	0.287	0.04	0.744	0.002	0.237	0.02	0.691	−13.60	<0.001	0.195

*Abbreviations:* *Adj. R*^*2*^ Adjusted R^2^ for the full model (block entry)

^a^Sex (coding: 0 for female; 1 for male)

^b^Age (years above 18) (age as continuous variable)

^c^Age (years above 18) quadratic term (age as continuous variable)

^d^Age-by-sex interaction (age (years above 18) as continuous variable)

^e^Health conditions (coding: 0 for no health condition; 1 for one or more health conditions)

For illustration purposes, the calculation of the predicted Global health status/QOL score for a 55-year-old French man with one or more health conditions based on the regression model is as follows:

$$\eqalign{{\text{Global health status/QOL }}\left( {{\text{predicted}}} \right)\, & {\text{ = }}76.78 + {\text{ sex}}\,*\,6.12{\text{ }} + {\text{ }}\left( {{\text{age}} - 18} \right){\text{ }}*{\text{ }} - 0.32{\text{ }} + {\text{ }}{\left( {{\text{age}} - 18} \right)^2} \cr & \quad *{\text{ }}0.01{\text{ }} + {\text{ }}\left( {{\text{age }} - {\text{ }}18} \right){\text{ }}*{\text{ sex }}*{\text{ }} - 0.09{\text{ }} + {\text{ health condition }}*{\text{ }} - 18.41 \cr}$$

$$\eqalign{{\text{Global health status/QOL }}\left( {{\text{predicted}}} \right){\text{ }}&= {\text{ }}76.78{\text{ }} + {\text{ }}1*{\text{ }}6.12{\text{ }} + {\text{ }}\left( {55 - 18} \right){\text{ }}*{\text{ }} - 0.32{\text{ }} + {\text{ }}{\left( {55 - 18} \right)^2} \cr &\quad *{\text{ }}0.01{\text{ }} + {\text{ }}\left( {55 - 18} \right){\text{ }}*{\text{ }}1{\text{ }}*{\text{ }} - 0.09{\text{ }} + {\text{ }}1{\text{ }}*{\text{ }} - 18.41{\text{ }} = {\text{ }}63.01 \cr}$$

## Discussion

In this study, we estimated general population normative data for the EORTC QLQ-C30 in the French general population. We present normative data separately for groups defined by sex, age, and presence of one or more chronic health conditions to support the interpretation of EORTC QLQ-C30 data in cancer research and clinical practice. To the best of our knowledge, this is the first study to present detailed normative data for this measure for France. In addition to descriptive general population normative data, the established regression models allow for ad hoc estimations of normative values as reference for cancer patient groups with specific sociodemographic and clinical characteristics.

In line with previous research, we found large differences in HRQoL between individuals with and without health conditions [17, 23], thus highlighting the detrimental impact of comorbidities on HRQoL. Additionally, our analysis provides a detailed insight into the impact of sex, age and comorbidities on HRQoL by stratifying for these factors. The impact of sex and age varied across the EORTC QLQ-C30 scales but was consistently much less pronounced than the impact of health conditions. Unlike in the descriptive (unadjusted) tables, in the multivariable regression models significantly better scores were found for men compared with women for Emotional Functioning, Cognitive Functioning, Insomnia and Fatigue whereas the opposite was true for Role Functioning and Financial Difficulties and Diarrhoea. In these models, Social Functioning, Global health status/QOL, Fatigue, Dyspnoea, Insomnia, and Financial Difficulties were significantly improved with increasing age. Interestingly, neither age nor sex showed a statistically significant association with the QLQ-C30 Summary Score in the multivariable regression model. In the Italian general population, older age was linked to improved HRQoL across several domains [19]. In contrast, the mixed sex and age patterns across the EORTC QLQ-C30 scales in this study were in line with results from the Austrian [18], German [17] and Spanish [20] general populations. The large impact of health conditions on HRQoL results has been previously reported for the general population [47–49]. Further, the impact of comorbidities was found in cancer patients and was reported for numerous populations, such as cancer survivors [47], elderly patients with cancer [48], patients with colorectal cancer [50], or patients with chronic myeloid leukaemia [51]. Most studies have simply focused on the impact of having a comorbidity and have not investigated in detail the impact of the type or number of comorbidities. In the Supplementary Table S3 we provide an additional regression analysis, which displays the incremental impact of the increasing number of comorbidities on HRQoL score. Similar to this finding, Park et al. showed that a higher number of comorbidities in breast cancer survivors was linked to lower PROMIS physical and mental HRQoL scores [52].

Such findings suggest the possible usefulness of adjusting for the presence of health conditions in patients with cancer when interpreting HRQoL data and comparing study populations. This may be particularly important when comparing data from effectiveness and efficacy trials, as populations that are eligible for trials can be highly selective and may exclude patients with comorbidities. When investigating country specific differences or general population norms Italian men almost exclusively reported better HRQoL scores compared to women [15], while Spanish women reported better HRQoL on several scales compared to Spanish men [20] which is similar to the pattern observed in the French population. Further, older-age was positively associated with HRQoL in Italy [19], whereby a German sample reported mixed age effect on EORTC QLQ-C30 scales [17]. While the impact of age also varies across various scales in the French population, higher age appears to be positively associated with better HRQoL, as indicated by the summary score. These country differences clearly show how crucial it is to present a detailed analysis of national norm data for each respective country included in the European Norm Data study, as these specific country differences might be missed if only overall data are shown. The French national general population norm data for the EORTC QLQ-C30 provide a very useful reference for the interpretation of HRQoL data as reported by French cancer patients.

General population norm data have been used to support the interpretation of HRQoL data from cancer clinical trials, including trials in patients with melanoma [53], multiple myeloma [54], and endometrial cancer [55]. Besides the interpretation of trial results, normative data have also been used to contextualise registry data, such as in the PROFILES registry [56] or the EORTC reference values dataset [26].

However, because patients with a large number of comorbidities are frequently excluded from randomized controlled trials [34], the evaluation of optimal treatment choices for this patient population remains limited, and the results of clinical trials that exclude this patient cohort may substantially overestimate the HRQoL experienced by patients. Knowledge about the impact of health conditions may help to translate findings from trial populations into daily clinical practice where patients with multimorbidity are much more common than in a trial setting. In clinical practice, patients with comorbidities may be subjected to deviation from treatment protocols or modification of potentially curative treatment owing to a lack of knowledge on how comorbidities interfere with cancer treatment [34]. Additionally, the presence of comorbidities may lead to concomitant treatments and polypharmacy in clinical practice [57], whereby differences in HRQoL results may also be influenced by varying toxicity profiles and side effects. This leads to the argument that real-world data with correctly interpreted HRQoL data are important for patients with pre-existing health issues.

Although there are various measures of comorbidity available (e.g., Adult Comorbidity Evaluation-27, Charlson Comorbidity Index, Chronic Disease Score, Cumulative Illness Rating Scale, Index of Coexisting Disease) [58], here, we relied on an ad-hoc assessment of self-reported comorbidities. The literature reports that sum scores of comorbidities result in loss of information. It is acknowledged that there is no gold standard in assessing comorbidities, but this is dependent on the context of the study [58]. The World Health Organisations’ EPIC study group recently investigated the aetiology and determinants of multimorbidity, which may inform the optimal assessment of comorbidities in patients with cancer [59].

A limitation of our study is the sampling procedure used when collecting responses of the general population. The panel research company GfK SE aims to provide representativeness of the general population; however, in the original study [16], although a high level of congruence between the study data and official population statistics was reported, highly educated individuals were overrepresented in the sample [60]. In a previous publication using this European dataset [45], higher education was found to be associated with higher HRQoL, although the impact was classified as small (the effect size eta^2^ was below 0.015 across all domains) [45]. Comorbidity data was collected via participant self-reported doctor-diagnosed chronic condition, whereby health conditions such as diabetes [61] and depression [62] appear to be slightly underrepresented in the current sample. The assessment of health conditions was aimed at covering common conditions with a possibly strong impact on HRQoL. However, it did not follow standardized assessment of comorbidities, such as using the Charlson Comorbidity Index [63], even though this may not be a suitable tool because it was designed to predict mortality rather than HRQoL impairment. Furthermore, the sampling procedure did not include strata for pre-existing health conditions, therefore the representativeness of this self-reported clinical variable is limited.

### Conclusion

To the best of our knowledge, this is the first study to provide French general population normative data for the EORTC QLQ-C30. The availability of general population normative data is useful to support the interpretation of HRQoL scores among French patients with cancer in clinical studies and clinical practice. Whilst the effects of sex and age varied across EORTC QLQ-C30 scales in the French population, there was a strong negative impact associated with the presence of comorbidities. These findings should be taken into consideration when interpreting HRQoL in patients with cancer. In conclusion, the present study presents new EORTC QLQ-C30 norm data from the French general population that can be used for comparative purposes with data obtained from French patients with cancer.

### Electronic supplementary material

Below is the link to the electronic supplementary material.

Supplementary Material 1

Supplementary Material 2

Supplementary Material 3

## Data Availability

The participants of this study did not give written consent for their data to be shared publicly, so due to the sensitive nature of the research supporting data is not available.
