# Supplementary material for: General population normative values for the EORTC QLQ-C30 by age, sex, and health condition for the French general population
Source: J Patient Rep Outcomes. 2024 May 2;8:48. doi: 10.1186/s41687-024-00719-7 (PMC11065800; doi:10.1186/s41687-024-00719-7)
Supplement: Supplementary file 3 — Supplementary Material 3 [file 41687_2024_719_MOESM3_ESM.docx]

Table S3: Regression models for the EORTC QLQ-C30 values, accounting for the incremental impact of the increasing numbers of health conditions, in the general population of France

|  | Intercept | Sex^1^ |  | Age^2^ |  | Age* squared^3^ |  | Age*-by-sex^4^ |  | One health condition^5^ |  | Two-to-three health conditions^5^ |  | Four or more health conditions^5^ |  |
| --- | --- | --- | --- | --- | --- | --- | --- | --- | --- | --- | --- | --- | --- | --- | --- |
|  |  | Coeff. | p-value | Coeff. | p-value | Coeff. | p-value | Coeff. | p-value | Coeff. | p-value |  | p-value |  | p-value |
| Physical Functioning | 95.31 | -3.37 | 0.128 | 0.127 | 0.341 | -0.003 | 0.118 | 0.136 | 0.018 | -7.95 | >0.001 | -15.78 | >0.001 | -30.90 | >0.001 |
| Role Functioning | 101.01 | -8.68 | 0.008 | -0.121 | 0.542 | 0.001 | 0.752 | 0.237 | 0.005 | -11.92 | >0.001 | -22.66 | >0.001 | -44.00 | >0.001 |
| Emotional Functioning | 67.21 | 9.61 | 0.006 | 0.400 | 0.058 | 0.001 | 0.730 | -0.079 | 0.380 | -8.79 | >0.001 | -21.43 | >0.001 | -33.27 | >0.001 |
| Cognitive Functioning | 86.50 | 3.80 | 0.195 | 0.213 | 0.231 | 0.000 | 0.860 | -0.083 | 0.273 | -8.39 | >0.001 | -15.31 | >0.001 | -30.45 | >0.001 |
| Social Functioning | 96.46 | -1.92 | 0.551 | -0.226 | 0.247 | 0.006 | 0.035 | 0.119 | 0.152 | -8.12 | >0.001 | -19.52 | >0.001 | -31.30 | >0.001 |
| Global QOL | 78.02 | 2.739 | 0.339 | -0.277 | 0.110 | 0.006 | 0.009 | -0.001 | 0.988 | -13.49 | >0.001 | -26.39 | >0.001 | -39.06 | >0.001 |
| Fatigue | 37.78 | -7.76 | 0.040 | -0.672 | 0.003 | 0.002 | 0.537 | 0.058 | 0.552 | 13.96 | >0.001 | 24.97 | >0.001 | 45.40 | >0.001 |
| Nausea / Vomiting | 3.46 | 3.03 | 0.108 | -0.028 | 0.810 | -0.001 | 0.581 | -0.096 | 0.049 | 2.89 | >0.001 | 4.42 | >0.001 | 13.53 | >0.001 |
| Pain | 7.02 | 4.26 | 0.246 | 0.090 | 0.684 | -0.001 | 0.698 | -0.194 | 0.041 | 15.33 | >0.001 | 28.35 | >0.001 | 51.22 | >0.001 |
| Dyspnoea | 10.48 | -2.94 | 0.433 | -0.235 | 0.301 | 0.003 | 0.428 | 0.074 | 0.445 | 8.89 | >0.001 | 20.61 | >0.001 | 39.33 | >0.001 |
| Insomnia | 19.83 | -8.94 | 0.057 | 0.526 | 0.065 | -0.013 | 0.001 | 0.034 | 0.778 | 11.57 | >0.001 | 26.01 | >0.001 | 45.09 | >0.001 |
| Appetite loss | 10.20 | -0.40 | 0.893 | -0.164 | 0.365 | 0.000 | 0.873 | -0.025 | 0.745 | 3.20 | 0.021 | 11.13 | >0.001 | 22.93 | >0.001 |
| Constipation | 10.09 | -1.33 | 0.700 | -0.090 | 0.665 | 0.002 | 0.582 | -0.119 | 0.183 | 6.06 | >0.001 | 10.25 | >0.001 | 15.07 | >0.001 |
| Diarrhoea | 4.84 | 5.39 | 0.069 | 0.021 | 0.908 | -0.001 | 0.559 | -0.136 | 0.077 | 1.91 | 0.163 | 9.06 | >0.001 | 17.89 | >0.001 |
| Financial Difficulties | -0.344 | 7.82 | 0.011 | 0.272 | 0.146 | -0.005 | 0.043 | -0.195 | 0.015 | 4.62 | 0.001 | 10.74 | >0.001 | 28.73 | >0.001 |
| QLQ-C30 Summary Score | 87.91 | 0.63 | 0.785 | 0.073 | 0.554 | 0.001 | 0.507 | 0.056 | 0.282 | -8.38 | >0.001 | -17.65 | >0.001 | -32.34 | >0.001 |
| 1 sex (coding: 0 for female; 1 for male)  2 age (years above 18) (age as continuous variable)  3 age (years above 18) quadratic term (age as continuous variable)  4 age-by-sex interaction (age (years above 18) as continuous variable)  5 health conditions (coding: 1 if the category applies (e.g., corresponding number of health conditions), 0 if the category does not apply) | | | | | | | | | | | | | | | |
